# Mapping the Olfactory Brain: A Systematic Review of Structural and Functional Magnetic Resonance Imaging Changes Following COVID-19 Smell Loss

**DOI:** 10.3390/brainsci15070690

**Published:** 2025-06-27

**Authors:** Hanani Abdul Manan, Rafaela de Jesus, Divesh Thaploo, Thomas Hummel

**Affiliations:** 1 Smell and Taste Clinic, Department of Otorhinolaryngology, Technische Universität Dresden, Fetscherstrasse 74, 01307 Dresden, Germany; rafaalexj@gmail.com (R.d.J.); taploodivesh4@gmail.com (D.T.); 2 Makmal Pemprosesan Imej Kefungsian (Functional Image Processing Laboratory), Department of Radiology, Universiti Kebangsaan Malaysia, Jalan Yaacob Latif, Bandar Tun Razak, Kuala Lumpur 56000, Malaysia; 3 Department of Radiology and Intervention, Hospital Pakar Kanak-Kanak (Children Specialist Hospital), Universiti Kebangsaan Malaysia, Jalan Yaacob Latif, Bandar Tun Razak, Kuala Lumpur 56000, Malaysia

**Keywords:** olfactory dysfunction, COVID-19’s SARS-CoV-2 smell loss, DTI, rs-fMRI, task-based fMRI, olfactory bulb volume, olfactory sulcus depth, brain connectivity

## Abstract

Background: Olfactory dysfunction (OD)—including anosmia and hyposmia—is a common and often persistent outcome of viral infections. This systematic review consolidates findings from structural and functional MRI studies to explore how COVID-19 SARS-CoV-2-induced smell loss alters the brain. Considerable heterogeneity was observed across studies, influenced by differences in methodology, population characteristics, imaging timelines, and OD classification. Methods: Following PRISMA guidelines, we conducted a systematic search of PubMed/MEDLINE, Scopus, and Web of Science to identify MRI-based studies examining COVID-19’s SARS-CoV-2 OD. Twenty-four studies were included and categorized based on imaging focus: (1) olfactory bulb (OB), (2) olfactory sulcus (OS), (3) grey and white matter changes, (4) task-based brain activation, and (5) resting-state functional connectivity. Demographic and imaging data were extracted and analyzed accordingly. Results: Structural imaging revealed consistent reductions in olfactory bulb volume (OBV) and olfactory sulcus depth (OSD), especially among individuals with OD persisting beyond three months, suggestive of inflammation and neurodegeneration in olfactory-associated regions like the orbitofrontal cortex and thalamus. Functional MRI studies showed increased connectivity in early-stage OD within regions such as the piriform and orbitofrontal cortices, possibly reflecting compensatory activity. In contrast, prolonged OD was associated with reduced activation and diminished connectivity, indicating a decline in olfactory processing capacity. Disruptions in the default mode network (DMN) and limbic areas further point to secondary cognitive and emotional effects. Diffusion tensor imaging (DTI) findings—such as decreased fractional anisotropy (FA) and increased mean diffusivity (MD)—highlight white matter microstructural compromise in individuals with long-term OD. Conclusions: COVID-19’s SARS-CoV-2 olfactory dysfunction is associated with a range of cerebral alterations that evolve with the duration and severity of smell loss. Persistent dysfunction correlates with greater neural damage, underscoring the need for longitudinal neuroimaging studies to better understand recovery dynamics and guide therapeutic strategies.

## 1. Introduction

The human sense of smell, though often understated, is fundamental to survival, influencing behaviors such as food intake, danger avoidance, and social interactions [1,2,3,4]. The brain regions responsible for olfactory processing, including the olfactory bulb, piriform cortex, orbitofrontal cortex, and amygdala, play crucial roles in sensory perception [5,6], emotional regulation [7], and memory formation [8].

The advent of neuroimaging, particularly magnetic resonance imaging (MRI) and functional MRI (fMRI), has revolutionized our ability to investigate the anatomy and functionality of the olfactory system in vivo. MRI provides high-resolution images of brain structures, allowing researchers to examine the volumetric and structural characteristics of olfactory regions and, in particular, olfactory bulbs [9,10,11,12]. In contrast, fMRI measures changes in blood oxygenation levels, enabling the visualization of brain activity in response to olfactory stimuli. These techniques together allow for a comprehensive examination of both the structural integrity and functional connectivity of olfactory pathways [13,14,15,16]. Importantly, MRI and fMRI have provided insights into neuroplasticity within olfactory circuits, developmental and age-related changes in olfactory processing, and the neural underpinnings of olfactory dysfunction in various clinical populations [17,18].

This systematic review aimed to synthesize current findings on COVID-19’s SARS-CoV-2 olfactory changes in the brain. COVID-19’s SARS-CoV-2 olfactory dysfunction has been linked to both structural and functional alterations, particularly involving the olfactory bulb, piriform cortex, and orbitofrontal cortex. COVID-19’s SARS-CoV-2 infections may damage the olfactory epithelium and disrupt neural connectivity, resulting in reduced olfactory bulb volume and altered activity within primary and secondary olfactory regions. These changes reflect impairments in odor processing, emotional integration, and memory. Neuroimaging studies consistently report hypoactivation and disrupted connectivity across key olfactory-related brain networks. Through a comprehensive review of literature from PubMed, Scopus, and Web of Science, this review sought to clarify the often fragmented and inconsistent findings across previous studies. The selected studies encompass a range of methodologies—including volumetric analysis, functional connectivity, and structural imaging—and examine both healthy individuals and clinical populations. Our objective was to consolidate current knowledge, resolve discrepancies in the literature, and provide a clearer framework to guide future research into the neural mechanisms underlying olfactory dysfunction following viral infections.

In this systematic review, we addressed several core aspects of olfactory neuroimaging research with a focus on post-viral alterations, particularly in the context of SARS-CoV-2 infection. First, we examined the anatomical characterization of olfactory brain regions and the relevance of volumetric MRI studies in identifying structural changes associated with olfactory capacity. Next, we explored how task-based (tb-fMRI) and resting-state fMRI (rs-fMRI) studies reveal the functional connectivity and neural dynamics of olfactory regions in response to odors and their associations with memory and emotion.

While previous systematic reviews [19,20] have examined olfactory dysfunction and imaging findings in broader or pre-pandemic populations, our review differs in both scope and timing. Specifically, we focused on MRI-based studies conducted in the aftermath of COVID-19, emphasizing neuroimaging techniques used to investigate both structural and functional correlates of post-viral olfactory impairment. Furthermore, we incorporated a broader range of imaging modalities—including DTI and multimodal approaches—to provide a more integrative understanding of how viral infections may affect the olfactory system at multiple levels of brain organization. Through this synthesis, we aimed to elucidate the extent to which diverse MRI methodologies can characterize the neural correlates of post-viral olfactory dysfunction—an area currently marked by heterogeneity in both the duration and manifestation of symptoms across studies. Despite these variations, our review integrated findings from structural, functional, and multimodal imaging to uncover previously unrecognized effects of olfactory impairment on brain plasticity. In doing so, we seek to advance the understanding of the broader cognitive, emotional, and neurological consequences associated with viral-induced disruptions to the olfactory system.

## 2. Materials

### 2.1. Search Strategy and Study Selection

To identify relevant studies, two independent researchers (Rafaela de Jesus and Divesh Thaploo) performed a comprehensive search across the PubMed/MEDLINE, Web of Science, and Scopus electronic databases. The search methodology followed the guidelines outlined in the Preferred Reporting Items for Systematic Reviews and Meta-Analyses (PRISMA), using a previously published study [21] as a reference for the search process. The objective was to find studies that reported structural alterations in the olfactory cortex associated with COVID-19, as reported in studies involving individuals with varying degrees of olfactory dysfunction or in comparison to healthy controls, as assessed using MRI. The search was conducted on 28 August 2024 and included articles published between 2020 and 2024 up to the search date. While this timeframe reflects a concentration of publications, it does not imply that olfactory research began during this period. Rather, the global impact of COVID-19 significantly accelerated interest and output in the field, bringing increased attention to post-viral olfactory dysfunction. The search terms used were: “(COVID OR corona) AND MRI AND (olfact * OR odor) AND human.” The asterisk (*) in the search term (olfact*) functions as a truncation operator, allowing for the retrieval of all word variants that share the same root. In this case, olfact* captures terms such as olfaction, olfactory, olfactometer, and olfactometry. This approach ensures a more comprehensive and inclusive search by encompassing multiple relevant terms derived from the same lexical stem, without the need to list each variant individually. The search strategy prioritized neuroimaging-specific terms to reduce retrieval of irrelevant records, though this may have limited sensitivity to studies using broader terminology (e.g., ‘smell,’ ‘SARS-CoV-2’). This limitation was partially addressed through manual citation tracking to ensure comprehensive coverage.

**Figure 1:**
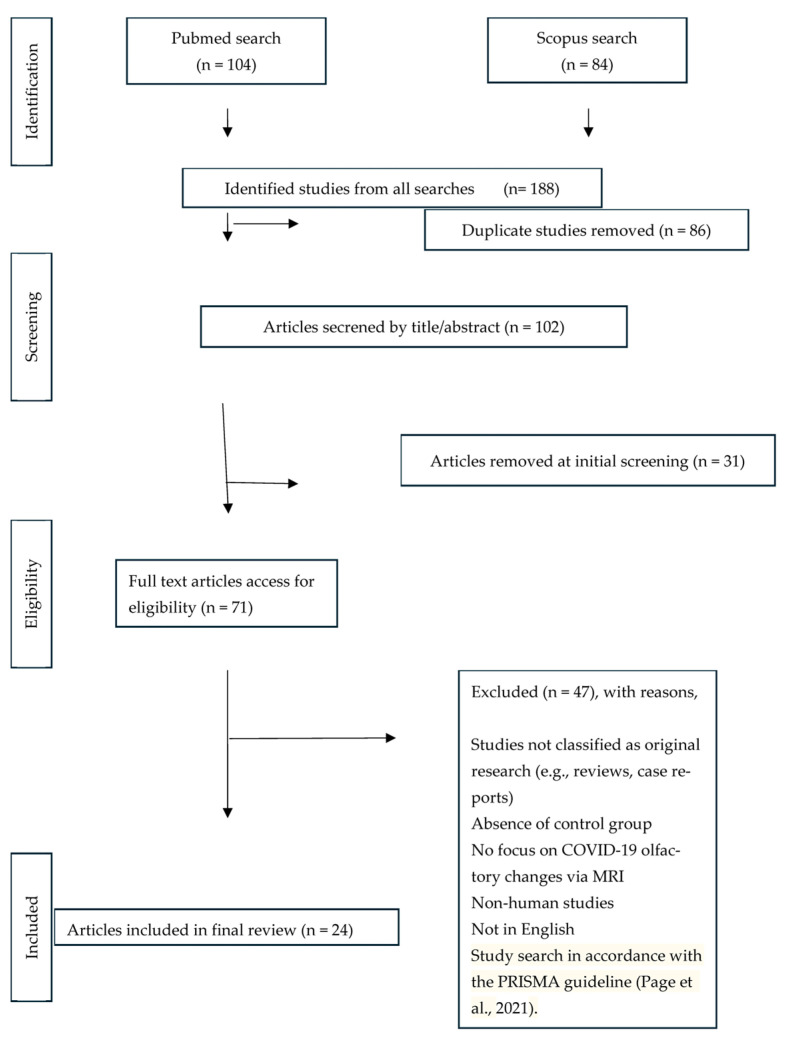
Study search following the PRISMA guideline (Page et al., 2021, [22]).

From all the articles retrieved, we removed duplicates to avoid bias. The decision to include search terms such as “COVID” or “corona” was made because, although many publications address post-viral smell loss more generally, they are often indexed or categorized specifically under COVID-related research. The titles and abstracts were initially screened independently by Rafaela de Jesus and Divesh Thaploo. This was followed by a full-text review to determine eligibility, with details of the included studies summarized in Figure 1. Any discrepancies regarding the eligibility of studies were resolved through discussion until a consensus was achieved.

### 2.2. Inclusion and Exclusion Criteria

To be included in this review, studies had to meet several criteria. Eligible studies were original, peer-reviewed research articles involving human participants with a confirmed diagnosis of COVID-19. To be included, studies must have employed magnetic resonance imaging (MRI) to evaluate structural or functional changes in the olfactory bulb, olfactory cortex, or other brain regions involved in olfactory processing. In addition to a confirmed COVID-19 diagnosis, participants in the patient group were required to exhibit COVID-19-related olfactory dysfunction, such as anosmia or hyposmia, as reported through clinical assessment or self-report. Studies that included COVID-19 participants without any documented olfactory symptoms were excluded from the final synthesis unless subgroup data specific to olfactory dysfunction were reported. Furthermore, studies were required to include a comparison group—either healthy controls or COVID-19 participants without olfactory impairment—to allow for meaningful between-group comparisons. These criteria were applied consistently during study selection and data extraction to ensure alignment with the review’s primary objective of investigating post-COVID olfactory-related brain changes.

Only studies published in English were considered to ensure consistency in data interpretation and analysis. Studies were excluded if they were not original research articles, such as systematic reviews, meta-analyses, case reports, or case series. Articles were also excluded if they lacked a control group or did not specifically focus on COVID-19-related alterations in olfactory structures using MRI.

The Population, Intervention, Comparison, Outcomes, and Study Design (PICOS) framework is tabulated in Table 1. We followed the PICOS framework, and the included studies primarily investigated populations comprising individuals with a history of moderate to severe SARS-CoV-2 infection, with or without persistent olfactory or cognitive symptoms. The interventions involved neuroimaging assessments using various MRI-based modalities, with comparisons typically made against healthy control groups or between symptomatic and asymptomatic post-COVID cohorts. The outcomes of interest were structural and functional alterations in brain regions associated with olfactory and cognitive processing. Most studies employed observational designs, either cross-sectional or longitudinal.

The neuroimaging techniques used across the studies included rs-fMRI, tb-fMRI, T1-weighted structural MRI, and diffusion tensor imaging (DTI). Rs-fMRI was utilized to assess functional connectivity between brain regions during rest, providing insight into intrinsic network alterations following infection. Tb-fMRI, on the other hand, was employed to evaluate brain activation in response to specific stimuli, such as olfactory cues or cognitive tasks, thereby enabling the assessment of stimulus-evoked neural responses. T1-weighted MRI was used to examine brain anatomy, including measurements of olfactory bulb volume and cortical thickness, which are critical structural markers in olfactory dysfunction. Finally, DTI was applied to investigate white matter integrity by quantifying the diffusion of water molecules along axonal pathways, particularly within the olfactory tracts and associated brain regions, to detect microstructural alterations that may underlie functional impairments.

In the included studies, the assessment of olfactory dysfunction (anosmia or hyposmia) varied in terms of methodology. The majority of studies relied on subjective self-report or patient recall to determine the presence and severity of olfactory loss, typically obtained through questionnaires or clinical interviews. Only a limited number of studies were incorporated. Psychophysical evaluations (e.g., Sniffin’ Sticks or University of Pennsylvania Smell Identification Test) to quantify olfactory performance. This inconsistency in olfactory assessment methods across studies represents a potential source of heterogeneity in the interpretation of results. Subjective assessments are prone to recall bias and may underestimate or overestimate dysfunction, whereas psychophysical methods provide a more standardized and reliable measure. The lack of uniformity in olfactory testing approaches should be considered when evaluating the strength of associations between imaging findings and olfactory status.

### 2.3. Bias and Quality Assessment

Given the methodological heterogeneity across included studies, formal risk of bias or quality assessment was not feasible. This limits the ability to systematically account for confounding or publication bias, and such potential sources of bias should be taken into consideration when interpreting the synthesized findings.

## 3. Results

### 3.1. Demographic Information

Twenty-four studies were selected for this review; there were a total of 1602 patients with anosmia or hyposmia and 1193 control participants. The age of participants varied from 18 to 81 years. The smallest sample size reported for patients with anosmia or hyposmia was 12 [23], while the largest sample size was 401 [24]. For controls, the smallest sample size was also 12, and the largest was 384 [23]. Of the 24 studies, nine utilized MRI (T1 or T2 image) alone to investigate olfactory dysfunction in COVID-19 patients. Some of the studies report a combination of techniques. Eight studies employed rs-fMRI, and two studies used tb-fMRI to assess brain activity related to olfactory function. Additionally, five studies incorporated diffusion tensor imaging (DTI) to evaluate microstructural brain changes associated with olfactory dysfunction. The details of the demographics of the selected studies are in Table 2.

### 3.2. Studies Using MRI Reported Olfactory Bulb Volume (OBV) and Olfactory Sulcus Depth (OSD)

Ten studies measured olfactory bulb volume (OBV) and olfactory sulcus depth (OSD), comprising 350 COVID-19 patients with olfactory dysfunction (OD) and 447 healthy controls. The age of participants ranged from 18 to 81 years old, with some studies reporting a more focused age range. For example, one study reported a median age of 53 years in COVID-19 patients and 55 years in controls [38], while another study with a younger cohort reported average ages of 26.6 ± 5.0 and 25.9 ± 2.8 years, respectively [34]. Across the studies, the groups were well matched for age and gender. Across studies, consistent structural and functional alterations were observed in olfactory-related brain regions—most notably the olfactory bulb, orbitofrontal cortex, and thalamus—despite methodological heterogeneity. Although differences in scanner field strength, gadolinium administration, and pulse sequences were present, these protocol-level variations did not obscure the overarching neuroanatomical patterns reported.

### 3.3. Olfactory Bulb Volume (OBV)

**Figure 2:**
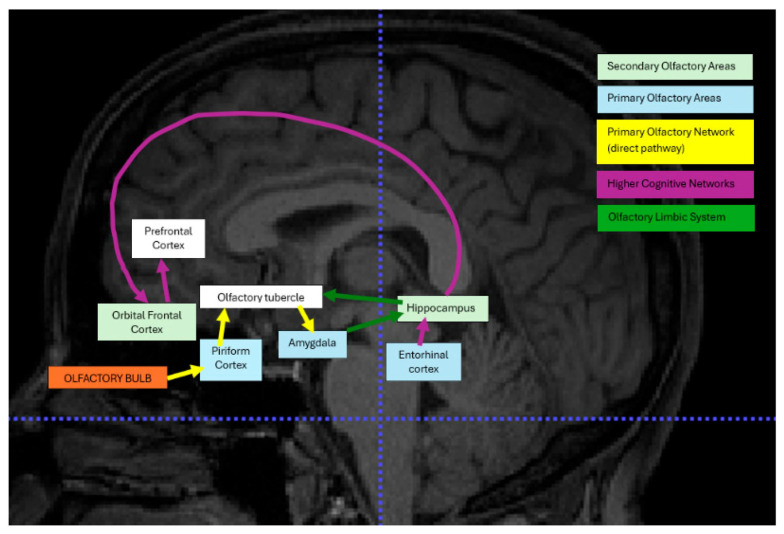
This visual presentation shows changes in functional brain connectivity that were reported in the selected studies in COVID-19 patients.

OBV was consistently lower in COVID-19 patients compared to controls; the details are tabulated in Figure 2. The size range for OBV in the COVID-19 group varied across studies (Table 3), from 37.1 ± 8.4 mm^3^ to 68.0 ± 14.3 mm^3^, while the control group OBV ranged from 49.1 ± 13.5 mm^3^ to 94.2 ± 7.6 mm^3^. Out of the ten studies reviewed, seven reported significantly smaller OBV among COVID-19 patients relative to controls. For example, one study found that the mean OBV in COVID-19 patients was significantly lower (68.0 ± 14.3 mm^3^) compared with the control group (94.2 ± 7.6 mm^3^); three of their patients were anosmic, and 38 were hyposmic [26]. Similarly, another study observed a marked reduction in OBV in COVID-19 patients for both the right (38 ± 8.5 mm^3^) and left (37.1 ± 8.4 mm^3^) sides compared with controls (56.3 ± 17.1 mm^3^ and 49.1 ± 13.5 mm^3^, respectively), with 67.7% of the patients being anosmic and the rest hyposmic [32]. In that study, the duration of olfactory loss ranged from three weeks to two months. Another study also reported a decrease in OBV, along with a wide variation in the duration of olfactory loss, ranging from 1 to 582 days; however, the type of olfactory dysfunction (OD) was not specified [38]. That study found the left OBV in patients to be significantly lower than in the control group. Other studies similarly confirmed reduced OBV in COVID-19 patients [28], although the OD types were not reported, supporting the notion that OD in these individuals correlates with structural alterations in the olfactory system. Another study reported significantly reduced OBV in COVID-19 patients (52.3 ± 13.6 mm^3^) compared with healthy controls (61.0 ± 15.8 mm^3^); participants exhibited anosmia and hypogeusia, with a reported mean duration of olfactory dysfunction of approximately 60 days [34]. Similarly, one study observed a reduction in OBV among COVID-19 patients [40]. However, the reported mean “disease duration” of 2.8 ± 2.4 days in that study likely refers to the acute phase of COVID-19 rather than the duration of olfactory dysfunction. This interpretation is supported by the broader literature, which consistently reports longer durations of COVID-19-related olfactory loss. A mean olfactory dysfunction duration of fewer than three days would be considered atypical and is not supported by current evidence. Thus, while that study contributes to evidence of OBV reduction following SARS-CoV-2 infection, the precise duration of olfactory impairment in their cohort remains unspecified.

In contrast, two studies reported no significant difference in OBV between COVID-19 patients and controls. One study found no significant variation in OBV between anosmic patients and controls but did not provide precise timing details regarding when the MRI was conducted relative to the COVID-19 diagnosis or the onset of anosmia, which could be a factor in their results [31]. Meanwhile, another study reported no notable difference in OBV between groups, even though the olfactory loss duration in their cohort ranged from 2 to 19 months, with patients presenting hyposmia and only one case of anosmia [18]. These findings suggest that the timing of MRI scans and the duration of olfactory loss may play critical roles in the variability of observed OBV reductions across different studies.

Interestingly, one study reported higher OBV in COVID-19 patients with olfactory dysfunction compared with controls, along with an association with olfactory cleft edema [36]. However, this study did not report the duration of olfactory loss. In conclusion, while most studies indicate a reduction in OBV in COVID-19 patients, two studies found no significant difference, and one study reported an increase in OBV.

### 3.4. Olfactory Sulcus Depth (OSD)

Olfactory sulcus depth (OSD) also showed a marked reduction in COVID-19 patients; details of the OSD are tabulated in Table 4. The OSD size range for patients spanned from 7.4 ± 0.1 mm to 7.98 ± 0.37 mm, while controls had OSD values ranging from 8.82 ± 0.74 mm to 9.6 ± 0.8 mm. Six studies reported a significant reduction in OSD among COVID-19 patients compared with controls. One study found that the mean OSD in the COVID-19 group was significantly lower (7.98 ± 0.37 mm) than in the control group (8.82 ± 0.74 mm) [26]. Similarly, another study observed a reduction in OSD in both the right (7.4 ± 0.1 mm) and left (7.4 ± 1.0 mm) sides in COVID-19 patients compared with controls (9.6 ± 0.8 mm and 9.4 ± 0.8 mm, respectively) [32]. In that study, olfactory loss lasted from three weeks to two months. Another study also found significantly lower OSD values in COVID-19 patients, with a reported mean olfactory loss duration ranging from 14 to 56 days [28]. One study reported cortical thinning in olfactory-related brain regions, contributing to reduced OSD in patients with anosmia, with a 60-day duration of olfactory loss [34]. Additionally, decreased OSD was observed in patients with increased disease severity and antiviral treatment duration [40]. Finally, another study reported reduced OSD in patients with olfactory dysfunction, where olfactory loss ranged from 1 to 582 days [38].

In contrast, two studies found no significant difference in OSD between COVID-19 patients and controls. One study reported no notable variation in OSD, while another did not observe significant differences in OSD between anosmic patients and controls [18,31]. However, one study reported an increase in OSD in COVID-19 patients with olfactory dysfunction, which was associated with olfactory cleft edema [36]. All three studies did not report the duration of olfactory loss. In summary, the majority of studies demonstrated reduced OSD in COVID-19 patients, while two studies reported no significant differences, and one study found higher OSD due to associated olfactory cleft conditions. Across studies, these reductions in OSD were consistent, highlighting the structural impact of COVID-19 on the olfactory system.

### 3.5. Studies Reported MRI on Grey Matter and White Matter Changes

Four studies examined brain structure changes following COVID-19 infection; details of the findings are tabulated in Table 5. A common finding across all studies is the observation of structural brain changes in COVID-19 patients, particularly those involving grey matter (GM). Each study reported either a reduction or alteration in grey matter, with varying degrees of severity based on factors like symptom burden and time since infection. In addition, olfactory-related brain regions were affected in three of the studies. For instance, one study failed to report the types of OD, and another reported on patients with anosmia and hypogeusia; both studies reported tissue damage or reductions in areas connected to the primary olfactory cortex [24,34], while a third study observed grey matter volume (GMV) changes in secondary olfactory areas [42]. The involvement of olfactory regions suggests that COVID-19, particularly in cases with anosmia, has a notable impact on brain structures related to the sense of smell. Ref. [24] reported that the mean duration of olfactory loss in their cohort was 141 days, while Ref. [34] observed olfactory loss lasting for 60 days. Furthermore, Ref. [42] reported a wider range of olfactory dysfunction duration, with patients experiencing olfactory loss between 2 and 16 months. These differences in the duration of olfactory dysfunction may contribute to the variation in the severity and type of brain structural changes observed across the studies.

Despite these similarities, the studies differ in patient demographics and the extent of brain changes observed. For example, Ref. [24] focused on an older population from the UK Biobank (aged 51–81 years), while Ref. [34] studied a younger cohort (average age 27 years). This age difference may explain the greater global brain size reduction observed in [24], as older individuals are more susceptible to age-related brain changes. Ref. [38], on the other hand, found that brain volume differences in their study were largely attributed to the higher proportion of females in the COVID-19 group, who naturally have smaller brain volumes than males. Furthermore, while Ref. [24] and Ref. [34] reported significant grey matter reductions, Ref. [42] found increased GMV in certain brain regions, particularly in long-COVID patients with neuropsychiatric symptoms. This suggests that the type of COVID-19 symptoms may influence whether brain volume increases or decreases in specific regions. For instance, Ref. [42] observed increased GMV in the limbic system, including the hippocampus and amygdala, in long-COVID patients with neuropsychiatric symptoms, whereas Ref. [24] reported significant reductions in the orbitofrontal cortex and parahippocampal gyrus, particularly in patients with anosmia. These findings highlight how distinct symptoms may correlate with structural changes in different brain areas.

### 3.6. Studies Reported Resting State-fMRI (rs-fMRI)

Eight studies investigated COVID-19-related olfactory dysfunction (OD) and cognitive impairment using resting state-fMRI (rs-fMRI). Table 6 tabulates the demographic information and important findings for these selected studies, and Figure 2 is the visual representation with labeled regions corresponding to the networks involved in the selected studies. These studies reported changes in both structural and functional brain connectivity within the olfactory network (ON) and related regions.

Most of the studies observed functional connectivity (FC) alterations, often noting increased connectivity in the olfactory cortex, such as the anterior piriform cortex, which is associated with compensatory mechanisms for olfactory loss [39]. Specifically, Ref. [39] found olfactory loss durations ranging from 29 to 93 days and reported on patients with anosmia and hyposmia, while Ref. [39] reported a shorter range of 0 to 21 days, and patients were hyposmic and hypogeusic. In addition to changes in the ON, altered FC was observed in other brain regions, such as the default mode network (DMN) and orbitofrontal cortex (OFC). Studies such as [43], who reported olfactory loss lasting between 91 to 268 days, and [37], who noted a mean duration of 168 ± 43 days for the anosmia group, correlated these FC changes with clinical measures of olfactory impairment. These findings underscore the broad impact of COVID-19-related OD on both olfactory-specific regions and wider functional brain networks. However, there are notable differences between the studies. The sample sizes, patient characteristics, and neuroimaging techniques varied considerably, with participant numbers ranging from small cohorts (e.g., 13 patients) to larger groups exceeding 100 participants. Furthermore, the regions showing FC alterations varied, with some studies emphasizing changes in the olfactory cortex [39] while others reported alterations in the hippocampus, insula, and cingulate cortex [8,37].

In summary, while current studies converge on the finding that COVID-19 alters neural connectivity associated with olfactory function, considerable variability remains regarding the specific structural changes and brain regions implicated. These discrepancies are likely attributable to differences in study design, patient demographics, symptom duration, and imaging modalities employed. Nonetheless, the overall body of evidence underlines the significant impact of COVID-19 on both olfactory processing and broader neural networks [18,35,39]. For instance, Ref. [35] reported reduced connectivity between the orbitofrontal cortex and the piriform cortex, two critical regions for olfactory perception and integration. Similarly, Ref. [18] identified disruptions in functional connectivity within the limbic system, particularly between the amygdala and hippocampus, regions essential for the emotional and memory-related components of olfactory experience. In addition, Ref. [39] observed altered connectivity within the default mode network (DMN), indicating that post-COVID olfactory dysfunction may also impact higher-order cognitive and sensory integration processes. Collectively, these findings highlight the multifaceted neural consequences of COVID-19-related olfactory impairment and emphasize the need for integrative approaches to studying its neurological sequelae.

### 3.7. Studies Reported Task-Based fMRI (tb-fMRI)

Two studies by Ref. [25] and Ref. [33] used task-based fMRI (tb-fMRI); details of the studies are tabulated in Table 7. These studies focus on the neural activation patterns related to olfactory dysfunction (OD), using different task-based approaches to assess brain regions involved in olfaction.

Both studies consistently observed altered brain activation in regions associated with olfactory processing across COVID-19 patients and other neurodegenerative conditions. Ref. [25], reporting on 14% anosmic and 86% hyposmic patients, revealed enhanced activity in the OFC and entorhinal cortices in COVID-19-related OD cases compared with post-infectious OD. These regions are critical for olfactory processing, and the study showed that trigeminal-sensory activity was more robust in COVID-19 patients, suggesting an intensified response to olfactory stimulation.

In contrast, Ref. [33] employed task-based fMRI to assess brain activation in COVID-19 patients with persistent olfactory dysfunction. Their patients were anosmic and microsmic. Their task involved olfactory stimulation, and they found significantly reduced activation in the upper frontal lobe and basal ganglia compared with healthy controls. The study showed that activation areas and the number of activations in these regions were notably lower, irrespective of the side of brain activation, pointing to a decline in the ability to process olfactory stimuli in patients with long-lasting olfactory impairment post-COVID. Ref. [33] reported that the mean duration of the olfactory loss is 3.13 ± 1.06 months.

Despite these similarities in the focus on olfactory-related brain regions, the studies differ in their specific tasks and populations. Ref. [25] compared COVID-19-related OD to post-infectious OD, using fMRI alongside MRI and DTI to measure responses to olfactory stimuli. Meanwhile, Ref. [33] focused solely on COVID-19 patients, employing olfactory stimulation tasks to study activation in the frontal and basal ganglia regions. In summary, both studies emphasize the impact of olfactory dysfunction on brain activation, particularly in regions such as the olfactory cortex (OFC) and basal ganglia, though they utilized different task-based fMRI methods to examine the distinct populations affected by OD.

### 3.8. Studies Reported Diffusion Tensor Imaging (DTI)

Six studies utilized diffusion tensor imaging (DTI) to examine microstructural changes in the brain, particularly focusing on the effects of COVID-19 on olfactory dysfunction and related neurological abnormalities. Details of the result are tabulated in Table 8.

All the studies reported alterations of DTI metrics in COVID-19 patients. Ref. [29] reported significant differences in fractional anisotropy (FA) and mean diffusivity (MD) between anosmic COVID-19 patients and controls in the olfactory bulb, with reduced FA and elevated MD values in COVID-19 patients. These findings were also supported by [25], who identified higher QA values in the orbitofrontal and entorhinal regions in COVID-19-related OD patients, indicating microstructural damage or reorganization in these regions; this study reported on anosmic and hyposmic patients. Additionally, Ref. [30] found reduced and altered structural connectivity in a subnetwork of parietal brain regions, alongside reduced local network efficiency in regions such as the left lateral orbital gyrus in COVID-19 patients with OD, further highlighting changes in brain networks associated with smell. Ref. [30] reported that the mean duration of the olfactory loss was 92 days (±26), ranging from 31 to 167 days, and patients were hyposmic.

However, differences emerge in the regions where DTI abnormalities are observed and their clinical correlates. Ref. [40] focused on the thalamus, insular gyrus, and amygdala in COVID-19 patients, finding significantly lower thalamus ADC values bilaterally, with no significant changes in the insular gyrus or amygdala. In contrast, Ref. [27] found significant changes in white matter (WM) integrity in regions like the superior frontal-occipital fasciculus (SFF) and corona radiata. Ref. [27] reported that the mean duration of the olfactory loss is 98 ± 8 days. Their DTI analysis revealed lower MD and higher FA in COVID-19 patients, indicating more subtle microstructural changes in white matter compared with the more pronounced differences in gray matter found in other studies. A unique aspect of the paper by [29] was the focus on defining diagnostic thresholds for FA and MD values to differentiate between normal and diseased olfactory bulbs, with FA and MD thresholds set at 0.22 and 1.5, respectively, and high sensitivity and specificity reported for these measures; the patients were anosmic. This contrasts with the more exploratory findings of [25], where structural changes in olfactory regions were noted but without specific diagnostic thresholds.

In summary, these studies converge on the finding that COVID-19 induces microstructural changes detectable via DTI in regions related to olfactory processing, such as the olfactory bulb, orbitofrontal cortex, and thalamus. However, they differ in their focus on specific brain regions, with some emphasizing diagnostic markers [29] and others exploring broader structural connectivity changes [30]. Table 8 tabulates the FA and MD values for all the selected studies. Together, these studies underscore the importance of DTI in revealing the microstructural impact of COVID-19 on the brain, particularly concerning olfactory dysfunction.

### 3.9. Studies Reported T2-Weighted Imaging (T2WI) and Postcontrast 3D T2 FLAIR, Diffusion-Weighted Imaging (DWI) and 3D High-Resolution T1-Weighted Imaging (T1WI)

The studies Refs. [27,41], alongside [23], offer valuable insights into the neuroimaging characteristics of COVID-19 patients, specifically concerning olfactory dysfunction and broader neurological symptoms. Table 9 summarizes the important findings from the studies that employed T2-weighted imaging (T2WI), postcontrast 3D T2 FLAIR, diffusion-weighted imaging (DWI), and 3D high-resolution T1-weighted imaging (T1WI) to investigate brain changes. The findings highlight significant structural and functional alterations in regions related to olfaction and their relevance to post-viral olfactory dysfunction.

Details of findings are tabulated in Table 9. Previous study [23] compared T2-weighted imaging (T2WI) and postcontrast 3D T2 FLAIR sequences between COVID-19 patients and an anosmia control group. They found a significant difference in normalized OB T2 FLAIR signal intensity between COVID-19 patients and controls, with COVID-19 patients showing higher signal intensity, indicating distinct structural or inflammatory changes in the OB. Previous study [23] reported that the mean duration of the olfactory loss was 14 days, with ranges from 0 to 32 days.

Previous study [41] expanded on these findings by using MRI and diffusion-weighted imaging (DWI) to examine apparent diffusion coefficient (ADC) values in both white matter (WM) and gray matter (GM) regions in COVID-19 patients with hyposmia. The study showed that COVID-19 patients, particularly those with neurological disorders, had significantly elevated ADC values in both WM and several GM regions. This ADC alteration was especially pronounced in patients hospitalized for neurological symptoms and in those with severe conditions like cognitive disorders or encephalitis. The findings highlight that the degree of microstructural brain damage, as reflected by ADC changes, correlates with the severity of neurological symptoms, adding another layer to the understanding of COVID-19’s impact on brain health beyond olfactory dysfunction. Caroli et al. [41] found that hospitalized patients experienced olfactory loss for an average of 77 days, whereas non-hospitalized patients reported a longer duration, averaging 252 days from the onset of symptoms.

Lu et al. [27] used 3D high-resolution T1-weighted imaging (T1WI) to investigate the microstructural changes in COVID-19 patients. Lu et al. [27] reported that the mean duration of the olfactory loss is 97.46 ± 8.01 days. Their results indicated significant increases in gray matter volume (GMV) in key olfactory-related regions, such as the bilateral olfactory cortices and hippocampi. These findings align with [23], as they underscore the impact of COVID-19 on both olfactory structures and other brain regions responsible for higher-order functions, such as memory and emotion processing. Overall, the combination of these studies demonstrates that COVID-19-related neurological effects extend beyond olfactory dysfunction, impacting multiple brain regions and microstructural properties. While Strauss et al. [23] focused on olfactory-specific changes, Caroli et al. [41] and Lu et al. [27] reveal broader alterations in both GM and WM, emphasizing the systemic nature of COVID-19’s impact on the brain.

## 4. Discussion

This review provides a comprehensive synthesis of structural and functional brain changes associated with olfactory processing following post-COVID-19 olfactory dysfunction. Findings across studies revealed substantial heterogeneity, influenced by factors such as study population characteristics (e.g., age and geographical location), timing of imaging relative to infection onset, focus of investigation, analytical techniques, and experimental paradigms. Despite this variability, consistent neural alterations were observed in key olfactory-related regions, including the olfactory bulb, orbitofrontal cortex, and thalamus. These changes were more pronounced in individuals with persistent olfactory dysfunction lasting beyond three months, suggesting a potential relationship between symptom duration and the extent of central nervous system involvement. Furthermore, different types of olfactory impairment—such as anosmia, hyposmia, or hypogeusia—were associated with distinct neuroimaging patterns, highlighting the lasting impact of post-COVID-19 smell loss on both brain health and olfactory network integrity. While the imaging modalities were grouped according to technique (MRI, fMRI, DTI), we acknowledge that variability in scanner field strength, gadolinium usage, and pulse sequences across studies may have contributed to subtle differences in reported findings. Nevertheless, such protocol-level factors are unlikely to have substantially influenced the overarching neuroanatomical patterns identified in this review, particularly as most studies performed within-subject or between-group comparisons using consistent acquisition settings. Therefore, the observed trends in olfactory-related brain alterations can be interpreted as robust across methodological differences, reinforcing the evidence for central nervous system involvement in post-viral olfactory dysfunction.

### 4.1. Olfactory Bulb Volume (OBV) and Olfactory Sulcus Depth (OSD)

The selected studies consistently demonstrate that COVID-19 is associated with reduced OBV and OSD among patients experiencing OD. This study focuses on the structural impact of COVID-19 on olfactory system components and how alterations in OBV and OSD may correlate with the duration of olfactory impairment and types of OD.

#### 4.1.1. OBV Reduction in COVID-19 Patients

In seven out of ten studies, COVID-19 patients displayed significant reductions in OBV compared with controls. While early hypotheses attributed olfactory dysfunction to potential neuroinvasion by SARS-CoV-2, this theory has since been thoroughly refuted. Current evidence indicates that neuroinvasion in humans is extremely rare and is not responsible for olfactory dysfunction or downstream neural circuit changes [46,47,48,49]. Instead, the observed OBV reduction is more plausibly explained by olfactory deprivation and neural disuse secondary to peripheral damage at the level of the olfactory epithelium. SARS-CoV-2 preferentially targets and eliminates non-neuronal support cells (particularly sustentacular cells), which disrupts the epithelial integrity and reduces sensory input to the olfactory bulb. This mechanism may be different from that of the less well-investigated traditional post-viral olfactory loss, which, at least in some cases, has been attributed to nasal obstruction or true viral neuroinvasion by neurotropic viruses. As reviewed by [50], the COVID-19 pandemic may represent a distinct pathophysiological scenario compared to prior respiratory virus outbreaks.

Thus, the reduction in OBV in COVID-19 patients likely reflects a downstream consequence of transient or sustained olfactory input loss, rather than direct viral effects on central neural structures. This understanding aligns with previous studies and provides a more accurate and evidence-supported framework for SARS-CoV-2. Additionally, the outlier finding by [36], which showed increased OBV associated with olfactory cleft edema, suggests that in some cases, inflammation-related edema may temporarily increase OBV, underscoring the complex pathology of COVID-19-related OD. Inflammation-related edema in the OB is a significant factor influencing OBV following COVID-19 infection, which has been documented across several studies [32,36,38]. This edema has been hypothesized to arise from the immune response to viral invasion, leading to the release of cytokines and other inflammatory mediators that can diffuse into the olfactory system. As these inflammatory processes accumulate in the OB and surrounding regions, the tissue becomes swollen, increasing OBV temporarily. Studies using MRI have observed that in COVID-19 patients with OD, this inflammation can sometimes produce an initial increase in OBV, reflecting edema or swelling [10,28,36].

#### 4.1.2. OSD Reduction and Its Association with OBV and OD Type

Six studies reported significant reductions in OSD among COVID-19 patients, with variations linked to the type and duration of olfactory dysfunction. Studies [26] and. [32] found reduced OSD in patients with anosmia, suggesting that complete smell loss may drive greater reductions in OSD compared to milder impairments, such as hyposmia. For example, Parlak et al. [32] observed bilateral OSD reductions in anosmic patients, with olfactory dysfunction lasting between three weeks and two months. Perlaki et al. [34] extended these findings by associating reduced OSD with cortical thinning in olfactory-related brain regions, particularly relevant in patients with long-term anosmia and hypogeusia.

As with OBV, some studies [18,31] did not report significant differences in OSD, possibly due to differences in imaging timing post-diagnosis or heterogeneity in OD types across participants. Notably, Abdou et al. [36] reported increased OSD in COVID-19 patients with olfactory dysfunction, which was attributed to olfactory cleft edema. This finding highlights that changes in OBV and OSD may not follow a linear trajectory and that associated inflammation or edema may contribute to observed volumetric variations.

#### 4.1.3. Duration of Olfactory Loss as a Mediator in OBV and OSD Variability

The variability in OBV and OSD findings across studies could also stem from differences in the duration of olfactory loss. For example, studies like Capelli et al. [38] and Burulday et al. [44], which tracked patients with prolonged olfactory dysfunction, generally reported more substantial reductions in OBV and OSD. Conversely, studies with shorter or undefined follow-up durations [18,31] found no significant structural differences, indicating that OBV and OSD reductions might progress or stabilize over time, depending on individual recovery rates. Altogether, the findings underscore that both the timing of imaging relative to the COVID-19 infection and the duration of OD are crucial factors in understanding the extent of structural damage to the olfactory system in COVID-19 patients.

In summary, OBV and OSD reductions in COVID-19 patients with OD point to structural changes in the olfactory system that correlate with OD type and duration. While most studies found decreases in both OBV and OSD, the findings by [36] and those without significant volume changes suggest that variations in inflammation, edema, and imaging timing may influence the detection of these structural alterations. Further, longitudinal studies are needed to clarify how OBV and OSD respond to prolonged olfactory dysfunction and to inform strategies for rehabilitation in post-COVID-19 olfactory impairments.

### 4.2. The Effect of SARS-CoV-2 on Grey Matter and White Matter Changes

The findings across multiple studies confirm that COVID-19 infection is associated with notable structural brain changes, specifically in GM and WM. COVID-19 infection is associated with GM and WM changes, which correlate with the duration and type of olfactory dysfunction. Patients with prolonged anosmia or hypogeusia appear more prone to GM and WM reductions, especially in primary olfactory and associated cortical regions. Conversely, those with neuropsychiatric symptoms may experience increases in GM volume, indicating a complex interaction between COVID-19 symptoms and brain structure changes.

#### 4.2.1. Grey Matter Changes and Their Association with Olfactory Dysfunction

GM volume reduction was a prominent finding across studies, particularly in olfactory-related brain regions. Studies by Douaud et al. [24] and Perlaki et al. [34] identified reductions in GM in primary olfactory cortical regions, which were notably prominent in patients with prolonged anosmia and hypogeusia. For example Douaud et al. [24] reported a mean olfactory loss duration of 141 days, suggesting that extended anosmia may contribute to GM volume reductions in areas responsible for olfactory processing. Similarly, Perlaki et al. [34], who observed GM atrophy in a younger cohort with an average olfactory loss of 60 days, reinforced the connection between prolonged olfactory impairment and GM structural changes in the olfactory pathway. These studies did not report specific OD types; however, GM reduction was consistently more severe in patients with extensive olfactory dysfunction, supporting a link between the duration of olfactory symptoms and GM volume loss.

On the other hand, Besteher et al. [42] reported increased GM volume in secondary olfactory regions, particularly in long-COVID patients presenting neuropsychiatric symptoms. This could imply that the nature of COVID-19 symptoms, such as anosmia versus neuropsychiatric symptoms, plays a role in determining whether GM volume decreases or increases. These increases in GM volume, particularly in patients with neuropsychiatric symptoms, may represent a compensatory response or reflect neuroplastic changes induced by prolonged symptoms. The findings suggest that anosmia and prolonged olfactory loss are associated with structural reductions, while neuropsychiatric symptoms, especially in long-COVID patients, may lead to GMV increases in specific regions.

#### 4.2.2. White Matter Changes and COVID-19-Related Olfactory Dysfunction

WM integrity is also affected in COVID-19 patients, though fewer studies directly addressed its correlation with olfactory dysfunction. WM integrity is also affected in patients with COVID-19, although relatively few studies have examined its direct relationship with olfactory dysfunction. Douaud et al. [24], using longitudinal data from the UK Biobank cohort (aged 51–81), reported widespread WM reductions in post-COVID individuals, contributing to an overall decrease in brain volume. These findings suggest that age may exacerbate the susceptibility of WM structures to SARS-CoV-2-related injury, potentially due to pre-existing vulnerabilities in aging neural tissue. Disruptions in WM integrity could compromise the connectivity between olfactory-related cortical and subcortical regions, which is essential for efficient olfactory signal integration.

However, this age-dependent vulnerability has not been universally observed. Perlaki et al. [34], for instance, documented WM alterations in a younger cohort with significant post-COVID olfactory dysfunction, highlighting that WM degeneration may also occur independently of age. Notably, their findings suggest that the severity or duration of olfactory impairment may be a more direct driver of WM changes than chronological age alone. This apparent discrepancy underscores the complexity of COVID-19’s neurological impact and suggests the possibility of multiple, non-mutually exclusive mechanisms, where age may modulate the extent of damage in some populations, while in others, prolonged sensory deprivation or inflammatory responses may drive WM alterations regardless of age. Further studies directly comparing age groups and controlling for duration of dysfunction are warranted to clarify these differential patterns of vulnerability.

#### 4.2.3. Impact of Demographics and Symptom Profile on GM and WM Changes

The population studied and the symptom profile appear to affect GM and WM changes observed in COVID-19 patients. For instance, Douaud et al. [24] reported more pronounced brain volume reductions in an older cohort, highlighting age as a risk factor for extensive structural changes. Meanwhile, Capelli et al. [38] noted that differences in GM and WM were influenced by the higher proportion of females in their cohort, reflecting possible sex-related anatomical differences. Additionally, Besteher et al. [42] observed that neuropsychiatric symptoms, rather than anosmia alone, correlated with increased GMV in some regions, suggesting that symptom type could determine whether structural changes manifest as reductions or compensatory increases in brain volume.

### 4.3. The Effect of SARS-CoV-2 on Brain Activation and Connectivity

A closer examination revealed that the duration and type of olfactory impairment, whether anosmia, hyposmia, or hypogeusia, correspond to specific patterns of functional connectivity and brain activation. Both rs-fMRI and tb-fMRI reveal significant COVID-19-related disruptions in olfactory and broader neural networks, with specific patterns correlating to OD duration and type. Patients with shorter OD durations often exhibit increased FC in regions such as the OFC, piriform cortex, and amygdala, suggesting the engagement of compensatory mechanisms to maintain olfactory function. Additionally, task-based activation studies reveal heightened responses in the insula and anterior cingulate cortex, reflecting attempts to adapt to olfactory deficits. In contrast, patients with chronic anosmia show reduced activation in the primary olfactory cortex, including the piriform and entorhinal cortices, as well as decreased FC between the OFC and hippocampus. These changes suggest a gradual decline in olfactory processing efficiency over time. Furthermore, broader disruptions are observed in the DMN and limbic system, such as diminished connectivity in the precuneus and posterior cingulate cortex, which may indicate secondary effects on cognitive and emotional regulation related to prolonged olfactory impairment.

#### 4.3.1. Resting-State fMRI Observations in COVID-19-Related OD

Most studies investigating COVID-19-related OD using rs-fMRI found alterations in FC within the olfactory network (ON) and its connections with broader brain networks, such as the DMN and OFC. Notably, Esposito et al. [45] and Esposito et al. [39] observed that patients with varying olfactory loss durations—ranging from 0 to 93 days—showed increased FC in olfactory regions like the anterior piriform cortex. This increased FC suggests a compensatory mechanism for addressing olfactory signal disruption. Similarly, Zhang et al. [43] and Wingrove et al. [37] observed prolonged olfactory dysfunction (91–268 days and 168 ± 43 days, respectively) correlated with FC alterations in both the ON and non-olfactory regions, indicating how chronic anosmia and hyposmia may progressively impact brain connectivity. These findings reinforce the idea that prolonged olfactory impairment, especially anosmia, induces adaptive but extensive connectivity changes, potentially to compensate for diminished olfactory function and related sensory experiences. However, differences in FC patterns across studies suggest that the extent and location of alterations are influenced by study design, cohort characteristics, and imaging techniques. For example, Esposito et al. [39] highlighted FC increases in the olfactory cortex, while Wingrove et al. [37] found altered connectivity in the hippocampus, insula, and cingulate cortex, regions associated with memory and emotional processing. This variability points to the complex effects of COVID-19-related OD on neural connectivity and highlights how rs-fMRI changes may vary depending on olfactory loss duration and symptom type.

#### 4.3.2. Task-Based fMRI Observations and Brain Activation in COVID-19-Related OD

Tb-fMRI studies provide additional insights by examining brain activation during olfactory-specific tasks. Across studies, consistent alterations in activation patterns were observed in critical olfactory processing areas, including the orbitofrontal cortex, piriform cortex, and basal ganglia, although activation intensity and specific regions varied based on OD duration and type. Yildirim et al. [25], who examined anosmic and hyposmic COVID-19 patients, reported enhanced activity in the orbitofrontal and entorhinal cortices, suggesting intensified neural responses to compensate for impaired olfactory function. Iravani et al. [33], examining patients with persistent anosmia and microsmia, observed reduced activation in the frontal lobe and basal ganglia, especially in patients with extended olfactory loss lasting approximately 3.1 ± 1.1 months. This reduction underscores a possible decline in olfactory processing capabilities in patients with prolonged COVID-19-related OD, aligning with findings from rs-fMRI studies that associate chronic anosmia with connectivity alterations.

#### 4.3.3. Correlation Between rs-fMRI and tb-fMRI Findings on OD Duration and Type

The findings across rs-fMRI and tb-fMRI studies consistently demonstrate that the duration and type of OD influence both connectivity and activation patterns. Patients with shorter olfactory loss durations often show compensatory increases in FC or task-induced brain activation, particularly in primary olfactory regions, as demonstrated by Esposito et al. [39] and Yildirim et al. [25]. Conversely, patients with longer-lasting OD exhibit more widespread connectivity alterations and diminished task-based activation responses, as seen in the studies by Zhang et al. [43] and Iravani et al. [33]. The observation of reduced activation in task-based studies for patients with chronic OD suggests that prolonged sensory deprivation due to anosmia or hyposmia may reduce the brain’s capacity to process olfactory stimuli effectively.

### 4.4. DTI, T2WI, DWI, and 3D High-Resolution T1WI-Based Findings in Olfactory Dysfunction

In recent studies, DTI, T2-weighted imaging (T2WI), diffusion-weighted imaging (DWI), and 3D high-resolution T1-weighted imaging (T1WI) have been instrumental in understanding the neurological impact of COVID-19 on OD. These imaging techniques consistently reveal distinct microstructural changes in patients with COVID-19-related olfactory impairment, yet they show variation in findings based on the duration and type of OD.

#### 4.4.1. White Matter Tracts and Olfactory Dysfunction

Most DTI-based studies reported consistent microstructural changes in key olfactory regions. For example, Sherif et al. [29] observed significant differences in FA and MD in the olfactory bulb of COVID-19 patients with anosmia, finding reduced FA and elevated MD values, suggesting microstructural compromise in this region. Yildirim et al. [25] further confirmed such findings with elevated QA values in the orbitofrontal and entorhinal cortices of anosmic and hyposmia patients, emphasizing microstructural alterations in regions beyond the primary olfactory cortex. These alterations correlated with patients experiencing both short- and longer-term olfactory loss, as indicated by [30], where patients with a mean olfactory loss duration of 92 days showed reduced structural connectivity in parietal regions and lower local network efficiency. Such findings suggest that longer-lasting anosmia or hyposmia might be associated with more pronounced microstructural alterations, possibly due to ongoing olfactory deprivation and neural disuse.

#### 4.4.2. Structural Changes in White and Gray Matter Through DWI and T1WI

Studies using T2WI, DWI, and T1WI show a broader impact on both white and gray matter regions. Caroli et al. [41] found that COVID-19 patients with hyposmia showed significant alterations in ADC values, particularly in hospitalized patients with severe neurological symptoms, which could suggest a link between COVID-19 severity and the degree of olfactory impairment. This study highlighted that hospitalized patients had shorter olfactory loss durations (~77 days) than non-hospitalized patients, whose olfactory impairment averaged ~252 days. This discrepancy suggests that the severity and setting of COVID-19 could impact both the type and duration of OD, influencing the extent of detectable ADC changes.

Strauss et al. [23] reported increased T2 FLAIR signal intensity in the OB of patients with anosmia, particularly those with shorter OD durations (14 days on average). In contrast, Lu et al. [27] found more widespread GM volume changes, notably in the bilateral olfactory cortices and hippocampi, in patients with longer durations of olfactory loss, averaging 98 days. Together, these studies reveal a pattern wherein patients with longer-lasting OD exhibit more extensive structural and connectivity changes, possibly linked to the persistence of sensory deprivation and compensatory mechanisms over time.

#### 4.4.3. Correlating Imaging Findings with OD Duration and Type

The imaging results across these studies suggest a compelling relationship between OD type (anosmia vs. hyposmia) and the duration of olfactory loss with neuroanatomical changes. Patients experiencing shorter olfactory loss durations, often in the context of anosmia, tend to show localized changes in primary olfactory regions (e.g., olfactory bulb signal intensity). In contrast, those with longer durations or cases of hyposmia exhibit broader structural and functional alterations, affecting multiple brain networks, such as the orbitofrontal and parietal regions. Such findings are supported by Sherif et al. [29] and Lu et al. [27], who noted that long-term olfactory loss is likely associated with compensatory network alterations across multiple olfactory and higher-order cortical regions.

### 4.5. Brain Plasticity and Responses to Environmental Stress and External Stimuli: Implications for Post-COVID-19 Recovery

Neuroplasticity refers to the brain’s ability to reorganize itself by forming new neural connections in response to experiences, environmental factors, or injury. In addition to the structural changes observed following viral infections, such as COVID-19, there is a growing body of research highlighting the brain’s adaptive responses to various forms of environmental stress and external stimuli. These changes are particularly relevant for understanding post-COVID-19 recovery and the neurological consequences that some individuals experience following the infection.

Studies have shown that the brain is not a static organ but is constantly reshaping itself based on internal and external influences. For example, chronic stress—which may be exacerbated by the social, economic, and psychological impacts of the COVID-19 pandemic—can induce significant changes in brain structures, such as the hippocampus, prefrontal cortex, and amygdala. These regions are critical for memory, decision-making, and emotional regulation. Prolonged stress related to illness, isolation, and uncertainty can lead to dendritic retraction, synaptic loss, and changes in the volume of these regions, yet in some cases, it can also trigger compensatory mechanisms to enhance connectivity and restore function. This phenomenon, often referred to as stress-induced plasticity, suggests that the brain is capable of adapting to environmental pressures [51,52].

Moreover, COVID-19 itself and the physiological stress it induces have been associated with neurological complications that could further exacerbate brain changes. For instance, some studies have reported neuroinflammation and hypoxia as a result of the viral infection, which can damage neurons and alter brain connectivity. However, there is evidence to suggest that the brain can still exhibit compensatory plasticity even in the face of such challenges. This is seen in individuals who experience post-COVID syndrome (or “long COVID”), where cognitive dysfunction and neurological symptoms such as anosmia (loss of smell) persist long after the acute infection resolves. The brain’s ability to reorganize itself in response to these disruptions is a testament to its plasticity, although the nature and extent of these changes are still under investigation [53].

Further, exposure to sensory deprivation, such as the loss of smell that often accompanies COVID-19 infection, can also promote neuroplastic changes. Studies suggest that the brain reorganizes itself to compensate for lost sensory input, which may help individuals recover olfactory function over time. However, the extent and effectiveness of this reorganization may depend on factors like the severity of the infection, the individual’s age, and the presence of other comorbidities. Recent research has demonstrated that targeted interventions such as olfactory training can lead to functional changes in the brain, even when structural changes are not apparent. For instance, a study by [54] examining women with COVID-19-associated olfactory dysfunction showed functional, but not structural, neural adaptations following a period of olfactory training. This finding highlights the brain’s remarkable neuroplastic potential, particularly within sensory systems, and supports the notion that directed stimulation can facilitate central compensation mechanisms after peripheral damage.

In the broader context of COVID-19, the brain’s response to neuroinflammation and other virus-induced stressors can result in neuroplasticity, either enhancing recovery or contributing to maladaptive reorganization. While some individuals may experience functional recovery through neuroplastic changes, others may face persistent symptoms as a result of incomplete or maladaptive plasticity. The ability of the brain to reorganize itself in the face of both viral damage and the emotional and psychological stress brought on by the pandemic highlights the complexity of brain changes following COVID-19 infection. Understanding these dynamic processes is critical for interpreting the long-term effects of COVID-19 on brain structure and function, particularly concerning sensory processing systems like olfaction.

### 4.6. Potential Confounding by Undiagnosed Neurodegenerative Conditions

It is also important to consider that some of the observed neuroimaging changes may not be solely attributable to post-viral olfactory dysfunction. Olfactory loss is an established early feature of several neurodegenerative diseases, particularly Parkinson’s and Alzheimer’s disease. Given the limited stratification for comorbidities or prodromal neurological conditions in most of the included studies, especially those involving older adults, there remains a possibility that some imaging findings—such as volume reductions in the orbitofrontal cortex or medial temporal regions—may reflect early-stage neurodegeneration rather than direct sequelae of SARS-CoV-2 infection. This confounding factor underscores the need for future studies to incorporate detailed clinical and neurocognitive profiling, particularly in aging populations, to better disentangle the effects of COVID-19 from age-related or preclinical neurodegenerative processes.

### 4.7. Limitations and Future Directions

The inclusion of a wide age range in the present studies presents a potential limitation for direct comparisons. Brain structure and function, including olfactory processing, can vary significantly across the lifespan, with distinct developmental and age-related changes. For example, younger individuals may exhibit more robust olfactory function and greater neural plasticity, while older participants may experience age-related decline in both olfactory sensitivity and brain connectivity. These inherent differences complicate the ability to disentangle the effects of viral infections from natural age-related changes. To mitigate this limitation, future research should consider age-matched comparisons or include age as a covariate in statistical analyses to account for developmental differences. Additionally, subgroup analyses focusing on specific age ranges could help clarify how viral infections impact olfactory functioning at different life stages. However, such adjustments were not feasible in the present analysis due to variability in reporting across studies and the lack of access to individual participant data. Many of the included studies either did not provide detailed age distributions or reported wide age ranges without stratification, limiting our ability to control for age-related effects systematically.

Another limitation pertains to the lack of detailed reporting on comorbidities and underlying health conditions in both patient and control groups. The inclusion and exclusion criteria were inconsistently specified across studies, potentially introducing confounding variables. Comorbid conditions—such as neurological disorders, metabolic diseases, or psychiatric illnesses—can significantly influence olfactory processing and brain structure, thereby complicating the interpretation of post-viral olfactory changes. Furthermore, the absence of well-defined participant selection criteria limits the generalizability of findings, as variability in baseline health status may introduce uncontrolled heterogeneity. Additionally, differences in participant nationality and cultural background across studies may also contribute to variability in outcomes. While such cross-national differences may reflect variations in genetic, environmental, and healthcare-related factors that influence olfactory function and brain response, these factors could not be systematically analyzed due to limited and inconsistent demographic reporting. Acknowledging these limitations allows for a more cautious interpretation of the findings and underscores the need for future studies to incorporate standardized, comprehensive demographic and clinical data—including age, comorbidities, and nationality—to better isolate the effects of viral infections on brain and olfactory function. Moreover, most studies did not report whether participants received olfactory interventions (e.g., training, corticosteroids, or neuroprotective agents), making it difficult to distinguish natural recovery from treatment effects. Future studies should consider stratifying imaging findings by treatment status to clarify these contributions.

## 5. Conclusions

The present review highlights how neuroimaging modalities like rs-fMRI, tb-fMRI, DTI, DWI, and T2WI enhance our understanding of COVID-19-related olfactory dysfunction. The present work also reveals that the structural and microstructural impacts on the brain vary with both the duration and severity of olfactory loss, providing crucial insights into the potential for persistent neurological sequelae in COVID-19 patients [45]. The specific relationship between imaging findings and olfactory dysfunction underscores the relevance of considering OD type and duration when assessing COVID-19’s impact on brain health.

## Figures and Tables

**Table 1:** PICOS for the study selection.

Population	Adults Above 18 to 81 Years Old with Post-COVID-19-Related OD
Interventions	All types of MRI techniques (including rs-fMRI, tb-fMRI, DTI)
Comparison	COVID-19 patients (diagnosis confirmed; olfactory status based on clinical assessment or self-reporting, including OD: anosmia, hyposmia, or normosmia)
Outcome	OBV, OSD, brain activation, brain connectivity, white matter integrity changes
Study design	except for case reports, case series, all types of reviews, letters to the editor

Abbreviation: olfactory bulb volume (OBV), olfactory sulcus depth (OSD), magnetic resonance imaging (MRI), resting-state fMRI (rs-fMRI), task-based fMRI (tb-fMRI), diffusion tensor imaging (DTI), olfactory dysfunction (OD). The colour is to highlight it.

**Table 2:** Demographic information and all the important findings.

Study (Author, Year)	Number of Patients	Number of Controls	Mean Age (Age Range; In Years)	Male (%)	Imaging Techniques
Yildirim D et al., 2022 [25]	31 (100% anosmic)	97 (19% hyposmic, 81% anosmic)	42.6 ± 14.1(19–80 years) (both groups)	38%	MRI, tb-fMRI, and DTI
Güney B et al., 2021 –no access [26]	41	42	40.27 ± 14.5(–missing age range)	49% (patients)	MRI
Lu Y et al., 2020, [27]	60 (2 anosmic)	39	Patients: 44.10 ± 16.00 Controls: 45.88 ± 13.90	57%	MRI and DTI
Douaud G et al., 2022 [24]	401	384	Patients: 58.9 ± 7.0 Controls: 60.2 ± 7.4	43%	MRI
Altunisik E et al., 2021 [28]	36	80	Patients: 37.33 ± 7.38 Controls: 35.74 ± 8.38	46%	MRI
Strauss SB et al., 2020 [23]	12 (1 anosmic)	12 (7 anosmic, 2 phanthosmic, 3 hyposmic)	Patients: 58.0 ± 13.8 Controls: 58.25 ± 14.9	38%	MRI
Sherif F et al., 2022 [29]	62 (100% anosmic)	23	Patients: 16–83 Controls: 17–61	24%	MRI, rs-fMRI, and DTI
Bispo DDC et al., 2023 [30]	33 (50% hyposmic)	20	Patients: 36.4 ± 9.5 Controls: 39.3 ± 12.9	30%	MRI, rs-fMRI, and DTI
Akkaya H, 2021 [31]	59 (100% anosmic)	64	Patients: 54.5 (21–71) Controls: 55 (19–80)	54%	MRI
Parlak AE et al., 2024 [32]	31 (68% anosmic, 32% hyposmic)	35	Patients: 54 ± 13.8 Controls: 59.9 ± 17.4	Not significant	MRI
Iravani K et al., 2024 [33]	15	5	33.33 ± 1.53 (both groups)	60%	MRI, tb-fMRI
Perlaki G et al., 2024 [34]	38 (100% anosmic)	37	Patients: 26 (23–29.3) Controls: 25 (24–27.5)	37%	MRI
Arrigoni A et al., 2024 [35]	51 (35 OD, 16 CM) (42 anosmic)	14 (CRTL)	COVID-OD: 40 (31–53) COVID-CM: 56 CTRL: 62 (45–70)	32%	MRI, rs-fMRI and DTI
Abdou EHE et al., 2023 [36]	110	50	Patients: 31.82 ± 10.15 Controls: 35.14 ± 12.37	28%	MRI
Wingrove J et al., 2023 [37]	28 (100% anosmic)	29 (11 + 18)	CoV: 37.02 ± 9.08 Long: 52.25 ± 12.17 Recov: 50.79 ± 8.91 Young: 27.73 ± 1.87 Controls: 38.89 ± 11.39	32%	MRI, rs-fMRI
Capelli S et al., 2023 [38]	196	39	Patients: 53 (42–60) Controls: 55 (46–66)	46%	MRI
Esposito F et al., 2023 [39]	18	10	Patients: 38.7 Controls: 33.1	53%	MRI, rs-fMRI
Muccioli L et al., 2023 [18]	23 (1 anosmic, most hyposmic)	26	Patients: 37 ± 14 Controls: 38.5 ± 13.7	49%	MRI, rs-fMRI
Burulday V et al., 2023 [40]	27	27	Patients: 35.25 ± 13.99 Controls: 35.62 ± 13.47	Not significant	MRI
Caroli A et al., 2023 [41]	215 (84 hyposmic)	36	Patients: 48 (36–55) Controls: 52 [42–65]	57%	MRI
Besteher B et al., 2022 [42]	30	20	Patients: 47.5 ± 11.5 Controls: 42.95 ± 13.41	46%	MRI
Zhang H et al., 2022 [43]	24	13	Patients: 43.6 ± 14.0 Controls: 45.0 ± 13.2	38%	MRI, rs-fMRI
Esposito F et al., 2022 [39]	27	18	Patients: 40.0 ± 7.6 Controls: 36.0 ± 7.1	36%	MRI, rs-fMRI
Burulday V et al., 2022 [44]	23	23	Patients: 37.08 Controls: 36.82	59%	MRI

Abbreviation: healthy control (CRTL), magnetic resonance imaging (MRI), resting-state fMRI (rs-fMRI), task-based fMRI (tb-fMRI), diffusion tensor imaging (DTI). Footnote: As the majority of participants did not fulfill the predefined inclusion criteria for COVID-related olfactory dysfunction, the study is included here to provide a comprehensive overview of the available neuroimaging literature. This deviation from the inclusion criteria is explicitly acknowledged and has been taken into account in the interpretation and discussion of the findings. We have also highlighted this in our PICOS.

**Table 3:** Olfactory bulb volume (OBV) for the selected studies.

Study	COVID-19 Group OBV (mm^3^)	Control Group OBV (mm^3^)	Type of OD	Duration of OD
Güney et al. (2021) [26]	68.0 ± 14.3	94.2 ± 7.6	Anosmia (3), Hyposmia (38)	Not reported
Parlak et al. (2024) [32]	Right: 38.0 ± 8.5, Left: 37.1 ± 8.4	Right: 56.3 ± 17.1, Left: 49.1 ± 13.5	Anosmia (67.7%), Hy-posmia (rest)	3 weeks to 2 months
Capelli et al. (2023) [38]	Left: 40.2 ± 9.7	Left: 50.8 ± 11.3	Not reported	1 to 582 days
Altunisik et al. (2021) [28]	Reduced OBV	Not provided	Not reported	Not reported
Perlaki et al. (2024) [34]	52.3 ± 13.6	61.0 ± 15.8	Anosmia, Hypogeusia	~60 days
Burulday et al. (2022) [44]	45.6 ± 10.4	60.2 ± 12.7	Not reported	Not reported
Akkaya et al. (2021) [31]	No significant difference	Not provided	Anosmia	Timing of MRI not reported
Muccioli et al. (2023) [18]	No significant difference	Not provided	Hyposmia (majority), Anosmia (1)	2 to 19 months
Abdou et al. (2023) [36]	72.1 ± 15.2	65.4 ± 14.8	Not reported	Not reported

Abbreviation: Olfactory bulb volume (OBV) and olfactory dysfunction (OD).

**Table 4:** Olfactory sulcus depth (OSD) of the selected studies.

Study	COVID-19 Group OSD (mm)	Control Group OSD (mm)	Type of OD
Güney et al. (2021) [26]	7.98 ± 0.37	8.82 ± 0.74	Not reported
Parlak et al. (2024) [32]	Right: 7.4 ± 0.1, Left: 7.4 ± 1.0	Right: 9.6 ± 0.8, Left: 9.4 ± 0.8	Not reported
Altunisik et al. (2021) [28]	7.5 ± 0.6	8.9 ± 0.5	Not reported
Perlaki et al. (2024) [34]	7.6 ± 0.9	8.8 ± 0.7	Anosmia
Burulday et al. (2022) [44]	7.3 ± 0.4	8.7 ± 0.6	Not reported
Capelli et al. (2023) [38]	7.7 ± 0.5	8.9 ± 0.8	Not reported
Muccioli et al. (2023) [18]	No significant difference	Not provided	Hyposmia, Anosmia (1)
Akkaya et al. (2021) [31]	No significant difference	Not provided	Anosmia
Abdou et al. (2023) [36]	8.1 ± 0.3	7.9 ± 0.4	Not reported

Abbreviation: olfactory bulb volume (OBV) and olfactory dysfunction (OD).

**Table 5:** Details of the studies that reported MRI on grey matter and white matter changes.

Study	Sample Size	Duration of Olfactory Loss	Brain Regions Involved	Findings	Types of OD
Douaud et al. (2022) [24]	401 COVID-19 patients, 384 controls The older population (UK Biobank, 51–81 years	141 days	The orbitofrontal cortex, parahippocampal gyrus	Significant grey matter reduction in primary and secondary olfactory areas	Not specified
Perlaki et al. (2024) [34]	50 patients Younger cohort (average age: 27 years)	60 days	Primary olfactory cortex	Tissue damage/reduction in primary olfactory areas	Anosmia, hypogeusia
Besteher et al. (2022) [42]	45 patients A varied age group	2–16 months	Secondary olfactory areas, hippocampus, amygdala	Increased GMV in limbic regions (e.g., hippocampus, amygdala) in long-COVID patients with neuropsychiatric symptoms	Long-COVID, neuropsychiatric symptoms
Capelli et al. (2023) [38]	75 COVID-19 patients, 60 controls Higher proportion of females in COVID-19 group	Not reported	Global brain regions	Brain volume differences attributed to a higher proportion of females; smaller brain volumes observed	Not specified

Abbreviation: olfactory dysfunction (OD).

**Table 6:** The important findings for the studies that reported the resting state fMRI.

Study	Sample Size	Duration of OD	Regions/Tracts Investigated	Findings	Networks Involved
Esposito et al. (2023) [39]	21 patients	29–93 days	Anterior piriform cortex, olfactory cortex	Increased FC in the olfactory cortex as a compensatory mechanism for anosmia and hyposmia	ON, DMN
Esposito et al. (2022) [45]	18 patients	0–21 days	Olfactory cortex	Increased FC in olfactory regions, patients were hyposmic and hypogeusia	ON
Zhang et al. (2022) [43]	40 patients	91–268 days	Olfactory cortex, DMN, OFC	Altered FC in olfactory regions, associated with clinical measures of olfactory impairment	DMN, OFC
Wingrove et al. (2023) [37]	50 patients	168 ± 43 days	Hippocampus, insula, cingulate cortex	Altered FC in limbic regions correlated with olfactory impairment	Limbic System, ON
Arrigoni et al. (2024) [35]	100+ participants	Not specified	Orbitofrontal cortex, piriform cortex	Reduced connectivity between the orbitofrontal and piriform cortex	ON, OFC
Muccioli et al. (2023) [18]	13 patients	Not specified	Amygdala, hippocampus	Disruptions in limbic system FC are linked to emotional and memory-related olfactory perception.	Limbic System
Sherif et al. (2022) [29]	25 patients	Not specified	Olfactory bulb	Altered structural connectivity; reduced FA and elevated MD values in the olfactory bulb	ON
Bispo et al. (2023) [30]	30 patients	92 ± 26 days	Left lateral orbital gyrus, parietal regions	Reduced structural connectivity and local network efficiency in regions associated with smell	DMN, ON

Abbreviation: olfactory dysfunction (OD), functional connectivity (FC), olfactory network (ON), default mode network (DMN), orbitofrontal cortex (OFC), gray matter volume (GMV), orbitofrontal cortex (OFC), fractional anisotropy (FA) and mean diffusivity (MD).

**Table 7:** The important finding for the studies that reported the task-based fMRI.

Study	Population	Olfactory Dysfunction	Brain Regions Involved	Findings	Task
Yildirim et al. (2022) [25]	COVID-19 patients (14% anosmic, 86% hyposmic)	COVID-19-related OD vs. post-infectious OD	Olfactory cortex (OFC), Entorhinal cortex	Enhanced activity in OFC and entorhinal cortices in COVID-19-related OD cases. Trigemino-sensory activity is more robust in COVID-19 patients.	fMRI, olfactory stimuli task
Iravani et al. (2024) [33]	COVID-19 patients (anosmic and microsmic)	Persistent olfactory dysfunction post-COVID	Upper frontal lobe, Basal ganglia	Significantly reduced activation in the upper frontal lobe and basal ganglia compared to healthy controls. Reduced ability to process olfactory stimuli.	Tb-fMRI, olfactory stimuli task

Abbreviation: functional magnetic resonance imaging (fMRI), resting-state fMRI (rs-fMRI), task-based fMRI (tb-fMRI), and olfactory dysfunction (OD).

**Table 8:** The important findings from the studies that reported the DTI findings.

Study	DTI Metrics	Involved Regions/White Matter Tracts	Findings	Patient Group
Sherif et al. (2022) [29]	↓ FA, ↑ MD	Olfactory bulb	Reduced FA, elevated MD in olfactory bulb	Anosmic COVID-19 patients
Yildirim et al. (2022) [25]	↑ QA	Orbitofrontal cortex, entorhinal cortex	Microstructural damage/reorganization	Anosmic and hyposmic patients
Bispo et al. (2023) [30]	↓ Structural connectivity	Left lateral orbital gyrus, parietal brain regions	Reduced local network efficiency	Hyposmic patients (mean duration of olfactory loss: 92 ± 26 days)
Burulday et al. (2023) [40]	↓ ADC	Thalamus	Lower ADC bilaterally in the thalamus	COVID-19 patients
Lu et al. (2020) [27]	↑ FA, ↓ MD	Superior frontal-occipital fasciculus (SFF), corona radiata	Subtle microstructural WM changes	COVID-19 patients (mean duration of olfactory loss: 98 ± 8 days)
Lu et al. (2020) [27]	FA < 0.22, MD > 1.5	Olfactory bulb	Defined diagnostic thresholds for FA and MD	Anosmic patients
Yildirim et al. (2022) [25]	N/A	Olfactory regions	Structural changes were noted, but no diagnostic thresholds	Anosmic and hyposmic patients

Abbreviation: ↓: Decrease, ↑: Increase, quantitative anisotropy(QA), apparent diffusion coefficient(ADC), white matter (WM), fractional anisotropy (FA), and mean diffusivity (MD).

**Table 9:** Key Findings from the selected studies utilizing various imaging modalities.

Study	Population	Olfactory Dysfunction	Imaging Techniques	Brain Regions Involved	Findings	Duration of Olfactory Loss
Strauss et al. (2020) [23]	COVID-19 patients vs. anosmia control group	COVID-19-related anosmia (higher OB T2 FLAIR intensity)	T2-weighted imaging (T2WI), postcontrast 3D T2 FLAIR	OB	Higher T2 FLAIR signal intensity in OB in COVID-19 patients, indicating structural or inflammatory changes.	Mean 14 days, range 0–32 days.
Caroli et al. (2023) [41]	COVID-19 patients with hyposmia (hospitalized and non-hospitalized)	COVID-19-related hyposmia (with microstructural brain changes)	MRI, DWI, ADC	WM, GM	Elevated ADC values in WM and GM regions, especially in patients with neurological symptoms. Correlation between ADC changes and symptom severity.	Hospitalized: 77 days; Non-hospitalized: 252 days
Lu et al. (2020) [27]	COVID-19 patients	COVID-19-related olfactory dysfunction	3D high-resolution T1-weighted imaging (T1WI)	Olfactory cortices, Hippocampus	Increased gray matter volume in key olfactory-related regions (olfactory cortices, hippocampi).	Mean 97.46 ± 8.01 days

Abbreviation: olfactory bulb (OB), white matter (WM), gray matter (GM), diffusion-weighted imaging (DWI), apparent diffusion coefficient (ADC), magnetic resonance imaging (MRI).

## Data Availability

Not applicable.

## References

[1] Boesveldt S., Parma V. (2021). The importance of the olfactory system in human well-being, through nutrition and social behavior. Cell Tissue Res..

[2] Bratman G.N., Bembibre C., Daily G.C., Doty R.L., Hummel T., Jacobs L.F., Kahn P.H., Lashus C., Majid A., Miller J.D. (2024). Nature and human well-being: The olfactory pathway. Sci. Adv..

[3] Melis M., Tomassini Barbarossa I., Sollai G. (2023). The Implications of Taste and Olfaction in Nutrition and Health. Nutrients.

[4] Schäfer L., Schriever V.A., Croy I. (2021). Human olfactory dysfunction: Causes and consequences. Cell Tissue Res..

[5] Georgiopoulos C., Buechner M.A., Falkenburger B., Engström M., Hummel T., Haehner A. (2024). Differential connectivity of the posterior piriform cortex in Parkinson’s disease and postviral olfactory dysfunction: An fMRI study. Sci. Rep..

[6] Gottfried J.A. (2010). Central mechanisms of odour object perception. Nat. Rev. Neurosci..

[7] Kadohisa M. (2013). Effects of odor on emotion, with implications. Front. Syst. Neurosci..

[8] Tong M.T., Peace S.T., Cleland T.A. (2014). Properties and mechanisms of olfactory learning and memory. Front. Behav. Neurosci..

[9] Gellrich J., Han P., Manesse C., Betz A., Junghanns A., Raue C., Schriever V.A., Hummel T. (2018). Brain volume changes in hyposmic patients before and after olfactory training. Laryngoscope.

[10] Rombaux P., Duprez T., Hummel T. (2009). Olfactory bulb volume in the clinical assessment of olfactory dysfunction. Rhinology.

[11] Whitcroft K.L., Mancini L., Yousry T., Hummel T., Andrews P.J. (2023). Functional septorhinoplasty alters brain structure and function: Neuroanatomical correlates of olfactory dysfunction. Front. Allergy.

[12] Zahnert F., Kleinholdermann U., Belke M., Keil B., Menzler K., Pedrosa D.J., Timmermann L., Kircher T., Nenadić I., Knake S. (2024). The connectivity-based architecture of the human piriform cortex. Neuroimage.

[13] Iravani B., Peter M.G., Arshamian A., Olsson M.J., Hummel T., Kitzler H.H., Lundström J.N. (2021). Acquired olfactory loss alters functional connectivity and morphology. Sci. Rep..

[14] Ruser P., Koeppel C.J., Kitzler H.H., Hummel T., Croy I. (2021). Individual odor hedonic perception is coded in temporal joint network activity. Neuroimage.

[15] Thaploo D., Joshi A., Yilmaz E., Yildirim D., Altundag A., Hummel T. (2023). Functional connectivity patterns in parosmia. Behav. Brain Funct..

[16] Waymel A., Friedrich P., Bastian P.A., Forkel S.J., Thiebaut de Schotten M. (2020). Anchoring the human olfactory system within a functional gradient. Neuroimage.

[17] Ma Y., Jiang J., Wu Y., Xiong J., Lv H., Li J., Kuang H., Jiang X., Chen Y. (2023). Abnormal functional connectivity of the core olfactory network in patients with chronic rhinosinusitis accompanied by olfactory dysfunction. Front. Neurol..

[18] Muccioli L., Sighinolfi G., Mitolo M., Ferri L., Jane Rochat M., Pensato U., Taruffi L., Testa C., Masullo M., Cortelli P. (2023). Cognitive and functional connectivity impairment in post-COVID-19 olfactory dysfunction. Neuroimage Clin..

[19] Tan H.Q., Choi J.S., Choi Y.J., Lee J.C. (2022). A systematic review of olfactory dysfunction and its neuroimaging findings n COVID-19 patients. Laryngoscope.

[20] Mohammadi A., Dehghani A., Parsa A.B., Sepehry A.A. (2023). Structural and functional alterations in the brain associated with olfactory dysfunction in COVID-19 patients: A systematic review. Neuroradiology.

[21] Manan A.A., Yahya N., Idris Z., Manan H.A. (2022). The Utilization of Diffusion Tensor Imaging as an Image-Guided Tool in Brain Tumor Resection Surgery: A Systematic Review. Cancers.

[22] Page M.J., McKenzie J.E., Bossuyt P.M., Boutron I., Hoffmann T.C., Mulrow C.D., Shamseer L., Tetzlaff J.M., Akl E.A., Brennan S.E. (2021). The PRISMA 2020 statement: An updated guideline for reporting systematic reviews. BMJ.

[23] Strauss S.B., Lantos J.E., Heier L.A., Shatzkes D.R., Phillips C.D. (2020). Olfactory Bulb Signal Abnormality in Patients with COVID-19 Who Present with Neurologic Symptoms. AJNR Am. J. Neuroradiol..

[24] Douaud G., Lee S., Alfaro-Almagro F., Arthofer C., Wang C., McCarthy P., Lange F., Andersson J.L.R., Griffanti L., Duff E. (2022). SARS-CoV-2 is associated with changes in brain structure in UK Biobank. Nature.

[25] Yildirim D., Kandemirli S.G., Tekcan Sanli D.E., Akinci O., Altundag A. (2022). A Comparative Olfactory MRI, DTI and fMRI Study of COVID-19 Related Anosmia and Post Viral Olfactory Dysfunction. Acad. Radiol..

[26] Güney B., Bacaksızlar Sarı F., Özdemir M.Y., Çullu N., Doğan E., Togan T. (2021). Changes in olfactory bulbus volume and olfactory sulcus depth in the chronic period after COVID-19 infection. Acta Otolaryngol..

[27] Lu Y., Li X., Geng D., Mei N., Wu P.Y., Huang C.C., Jia T., Zhao Y., Wang D., Xiao A. (2020). Cerebral Micro-Structural Changes in COVID-19 Patients—An MRI-based 3-month Follow-up Study. EClinicalMedicine.

[28] Altunisik E., Baykan A.H., Sahin S., Aydin E., Erturk S.M. (2021). Quantitative Analysis of the Olfactory System in COVID-19: An MR Imaging Study. AJNR Am. J. Neuroradiol..

[29] Sherif F., Elmokadem A.H., Abdel Razek A., Kamal E., Abdou E.H.E., Salem M.A., Ghoneim M.M. (2022). DTI of the Olfactory Bulb in COVID-19-Related Anosmia: A Pilot Study. AJNR Am. J. Neuroradiol..

[30] Bispo D.D.C., Brandão P.R.P., Pereira D.A., Maluf F.B., Dias B.A., Paranhos H.R., von Glehn F., de Oliveira A.C.P., Soares A.A.S.M., Descoteaux M. (2023). Altered structural connectivity in olfactory disfunction after mild COVID-19 using probabilistic tractography. Sci. Rep..

[31] Akkaya H., Kizilog Lu A., Dilek O., Belibag Li C., Kaya Ö., Yılmaz C., Gülek B. (2021). Evaluation of the olfactory bulb volume and morphology in patients with coronavirus disease 2019: Can differences create predisposition to anosmia?. Rev. Assoc. Med. Bras. (1992).

[32] Parlak A.E., Selçuk Ö.T., Yilmaz G.Ö., Aydenizoz D., Selçuk N.T., Öcal R., Seyman D., Yilmaz M., Eyigör H. (2024). Olfactory Bulb Volume and Morphology Changes in COVID-19 Patients With Olfactory Disorders Using Magnetic Resonance Imaging. J. Comput. Assist. Tomogr..

[33] Iravani K., Malekpour B., Rasekhi A., Faramarzi A., Soltaniesmaeili A., Golkhar B., Jahanandish F., Babaei A. (2024). Functional magnetic resonance imaging in coronavirus disease 2019 induced olfactory dysfunction. J. Laryngol. Otol..

[34] Perlaki G., Darnai G., Arató Á., Alhour H.A., Szente A., Áfra E., Nagy S.A., Horváth R., Kovács N., Dóczi T. (2024). Gray Matter Changes Following Mild COVID-19: An MR Morphometric Study in Healthy Young People. J. Magn. Reson. Imaging.

[35] Arrigoni A., Previtali M., Bosticardo S., Pezzetti G., Poloni S., Capelli S., Napolitano A., Remuzzi A., Zangari R., Lorini F.L. (2024). Brain microstructure and connectivity in COVID-19 patients with olfactory or cognitive impairment. Neuroimage Clin..

[36] Abdou E.H.E., Ebada H.A., Salem M.A., Ghoneim M.M.R., Sherif F., Kamal E. (2023). Clinical and Imaging Evaluation of COVID-19-Related Olfactory Dysfunction. Am. J. Rhinol. Allergy.

[37] Wingrove J., Makaronidis J., Prados F., Kanber B., Yiannakas M.C., Magee C., Castellazzi G., Grandjean L., Golay X., Tur C. (2023). Aberrant olfactory network functional connectivity in people with olfactory dysfunction following COVID-19 infection: An exploratory, observational study. EClinicalMedicine.

[38] Capelli S., Caroli A., Barletta A., Arrigoni A., Napolitano A., Pezzetti G., Longhi L.G., Zangari R., Lorini F.L., Sessa M. (2023). MRI evidence of olfactory system alterations in patients with COVID-19 and neurological symptoms. J. Neurol..

[39] Esposito F., Cirillo M., De Micco R., Caiazzo G., Siciliano M., Russo A.G., Monari C., Coppola N., Tedeschi G., Tessitore A. (2023). Olfactory Loss and Brain Connectivity after COVID-19: Structural Follow-Up at One Year. Neural Plast..

[40] Burulday V., Bayar Muluk N., Akgül M.H., Sayar M.S. (2023). Diffusion-weighted imaging measurements of central smell regions in COVID-19 patients: Insular gyrus, corpus amygdala, and thalamus. Eur. Rev. Med. Pharmacol. Sci..

[41] Caroli A., Capelli S., Napolitano A., Cabrini G., Arrigoni A., Pezzetti G., Previtali M., Longhi L.G., Zangari R., Lorini F.L. (2023). Brain diffusion alterations in patients with COVID-19 pathology and neurological manifestations. Neuroimage Clin..

[42] Besteher B., Machnik M., Troll M., Toepffer A., Zerekidze A., Rocktäschel T., Heller C., Kikinis Z., Brodoehl S., Finke K. (2022). Larger gray matter volumes in neuropsychiatric long-COVID syndrome. Psychiatry Res..

[43] Zhang H., Chung T.W., Wong F.K., Hung I.F., Mak H.K. (2022). Changes in the Intranetwork and Internetwork Connectivity of the Default Mode Network and Olfactory Network in Patients with COVID-19 and Olfactory Dysfunction. Brain Sci..

[44] Burulday V., Yıldız S., Tuncer A., Atagün İ., Yiğit D., Gürkan H., Genç E., Alkan A. (2022). Structural brain changes in patients with long COVID: A voxel-based morphometry study. Brain Commun..

[45] Esposito F., Cirillo M., De Micco R., Caiazzo G., Siciliano M., Russo A.G., Monari C., Coppola N., Tedeschi G., Tessitore A. (2022). Olfactory loss and brain connectivity after COVID-19. Hum. Brain Mapp..

[46] Butowt R., von Bartheld C.S. (2021). Anosmia in COVID-19: Underlying Mechanisms and Assessment of an Olfactory Route to Brain Infection. Neuroscientist.

[47] Butowt R., Meunier N., Bryche B., von Bartheld C.S. (2021). The olfactory nerve is not a likely route to brain infection in COVID-19: A critical review of data from humans and animal models. Acta Neuropathol..

[48] Tsukahara T., Ueha R., Kondo K., Yamasoba T. (2023). Pathological findings of the olfactory mucosa in persistent COVID-19–related olfactory dysfunction. Laryngoscope.

[49] Meinhardt J., Radke J., Dittmayer C., Franz J., Thomas C., Mothes R., Laue M., Schneider J., Brünink S., Greuel S. (2021). Olfactory transmucosal SARS-CoV-2 invasion as a port of central nervous system entry in individuals with COVID-19. Nat. Neurosci..

[50] Butowt R., Bilinska K., von Bartheld C.S. (2023). Olfactory dysfunction in COVID-19: New insights into the underlying mechanisms. Trends Neurosci..

[51] McEwen B.S. (2007). Physiology and neurobiology of stress and adaptation: Central role of the brain. Physiol. Rev..

[52] Radley J.J., Sapolsky R.M. (2006). Stress-induced neuronal remodeling in the hippocampus: Implications for the pathophysiology of mood disorders. Neurobiol. Dis..

[53] Chou S.H.Y., Beghi E., Helms S. (2021). COVID-19 neurological complications and brain plasticity. Lancet Neurol..

[54] Li Z., Gebler J., Joshi A., Xu X., Thaploo D., Hähner A., Avaro V., Calegari F., Hummel T. (2025). Functional but Not Structural Brain Changes After Olfactory Training in Women with COVID-19-Associated Olfactory Dysfunction. Laryngoscope.

